# Assessment of the Body Composition and the Loss of Fat-Free Mass through Bioelectric Impedance Analysis in Patients Who Underwent Open Gastric Bypass

**DOI:** 10.1155/2014/843253

**Published:** 2014-01-09

**Authors:** Wilson Rodrigues de Freitas Junior, Elias Jirjoss Ilias, Paulo Kassab, Roberto Cordts, Paulo Gustavo Porto, Francisco Cesar Martins Rodrigues, Mohamed Ibrahim Ali Taha, Paulo Carrara, Isabella de Carvalho Aguiar, Luis Vicente Franco de Oliveira, Osvaldo Castro, Carlos Alberto Malheiros

**Affiliations:** ^1^Gastric Surgery Division, Department of Surgery of Santa Casa of São Paulo Medical School, São Paulo, SP, Brazil; ^2^Department of Epidemiology of Santa Casa of São Paulo Medical School, São Paulo, SP, Brazil; ^3^Master's and Doctoral Programs in Rehabilitation Sciences, Nove de Julho University (UNINOVE), São Paulo, SP, Brazil

## Abstract

*Background*. Bariatric surgery is considered an effective option for the management of morbid obesity. The incidence of obesity has been gradually increasing all over the world reaching epidemic proportions in some regions of the world. Obesity can cause a reduction of up to 22% in the life expectancy of morbidly obese patients. *Objective*. The objective of this paper is to assess the weight loss associated with the first 6 months after bariatric surgery using bioelectric impedance analysis (BIA) for the evaluation of fat mass and fat-free mass. *Method*. A total of 36 morbidly obese patients were subjected to open gastric bypass surgery. The patients weight was monitored before and after the procedure using the bioelectric impedance analysis. *Results*. Bariatric surgery resulted in an average percentage of weight loss of 28.6% (40 kg) as determined 6 months after the procedure was performed. Analysis of the different components of body weight indicated an undesirable loss of fat-free mass along with the reduction of total body weight. *Conclusion*. Open gastric bypass induced a significant loss of total weight and loss of fat-free mass in patients six months after the surgery. The use of bioelectric impedance analysis resulted in an appropriate estimation of the total weight components in individuals subjected to bariatric surgery allowing a more real analysis of the variation of weight after the surgery.

## 1. Introduction

Bariatric surgery was developed with the purpose of treating problems inherent in obesity, especially level II and III, which exhibited difficulty for conventional clinical treatments [1]. This type of surgery gained importance as one of the best alternatives for those specific groups since it is the only method that induces a large weight loss after the first year of treatment [2, 3].

Obesity is the second major cause of death, by avoidable causes, in the United States [4]. Annually, 300 thousand people die as a consequence of obesity-related problems and the risk of premature death among those with excess weight increases 1.5–2-fold when compared to individuals with BMI values between 20 and 25 kg/m^2^ [5, 6].

This epidemic occurs even in developing countries like Brazil, where it is estimated that there are 17 million obese people with incidence levels of 5.9% among adult men and 13.3% among adult women. A survey carried out in Brazil in 1996 showed that the incidence of overweight problems in Brazilian children was about 4.9% [7].

Obesity can cause a reduction of 22% in the life expectancy of morbidly obese people representing a reduction of up to 12 years in the life of a 25-year-old morbidly obese person [1]. On the other hand, we do not want to change the obesity problem for a malnutrition one.

This study aimed to assess the weight loss associated with the first 6 months after surgery of patients subjected to open Roux-en-Y gastric bypass procedure, using bioelectrical impedance analysis (BIA) for the evaluation of fat mass and fat-free mass.

## 2. Methods

A prospective study was carried out with patients subjected to bariatric surgery and conducted according to the ethical standards established in the 1961 Declaration of Helsinki (as revised in Hong Kong in 1989 and in Edinburgh, Scotland, in 2000). This study is related to protocol registered in the World Health Organization Universal Trial Number (UTN) U1111-1121-8873 and Registro Brasileiro de Ensaios Clínicos (RBR-9k9hhv) and has been approved by the Human Research Ethics Committees of the Nove de Julho University, São Paulo, Brazil (process number 220506/2009) [8]. All participants gave written informed consent. The sample was composed by 36 patients, severe obese, 29 females (80.6%), and 7 males, all between the ages of 25 and 56 years old. All patients were enrolled in the surgical treatment program of the Gastric Surgery Department of Santa Casa Hospital, São Paulo, Brazil. The preparation of the patients consisted in a thorough examination by different teams involving areas such as psychology, surgery, nutrition, clinical medicine, endocrinology, and anesthesiology.

Weight and height of all subjects were collected by the surgeon responsible for each case using an anthropometric electronic scale (Welmy Indústria e Comércio Ltda, model 200/5; São Paulo, Brazil) equipped with a ruler (±0.5 cm). The data was collected immediately before the procedure and 6 months after bariatric surgery.

All patients under analysis had been diagnosed with severe obesity and were recommended to have a surgical treatment since all of them fulfilled the criteria established by the International Federation for Surgery of Obesity (IFSO) and the Brazilian Health Department. The inclusion criteria were to have an BMI greater or equal to 40 kg/m^2^, or greater or equal to 35 kg/m^2^ with comorbidities related to obesity and considered obese for more than 5 years, who had already undergone clinical treatments in the last 2 years and aged 18 to 65 years old, and to have an acceptable surgical risk.

The selection of the patients was carried out consecutively excluding those who exhibited a condition that could interfere with the reliability of BIA such as alterations in the blood levels of sodium, potassium, and calcium; any evidence of renal dysfunction or failure; presence of any type of skin lesions on the areas where the electrodes were to be placed, and edema or heart failure which could contribute to an inflation of the extracellular compartment (Na and water inflation) that could explain an important decrease of lean mass after surgery.

We used the BIA of four channels, Quantum II Bioelectrical Body Composition Analyzer (RJL Systems, Detroit, MI, USA), which assessed the resistance and reactance allowing the determination of the different components of body weight such as fat-free mass, fat mass, and water content. Those values were calculated using the computer software V. Corp (RJL Systems, Detroit, MI, USA) taking into consideration height, weight, gender, age, and level of physical activity. This procedure was validated by some obesity research [9–11].

The BIA test measures the voltage produced between two electrodes located near the sites where the current is applied, and it is expressed as the ratio (*Z*) between voltage (*V*) and electrical current (*I*), according to the National Institute of Health (USA) [12]. The electrical current used is 800 *μ*A at a frequency of 50 khz, which is not perceived by human beings and it is not affected by interference from bioelectrical sources such as the muscles or the heart.

Abdominal incision was marked and started from 2 cm below xiphoid process to 7 cm above the umbilicus. Surgical procedures were of the type gastric bypass with reconstruction “Roux-en-Y” [13], with small pouch kind of Capella with gastrointestinal anastomosis in two sutures, being one of continuous 4-0 vicryl and other seromuscular cotton 3-0 with sutures, with lateral anastomosis 1.5 cm in diameter, and placement of ring silastic. The loop food is 100.0 cm and the handle is 50.0 cm biliopancreatic with enteroanastomosis lateral side 3-0 vicryl continuous in 2 planes with a diameter of 4.0 cm. The surgical procedures were performed by 2 surgeons who alternated between surgeon and first assistant to each surgery.

With the purpose of performing a more appropriate analysis, all patients were required to comply with several conditions prior to the BIA such as no food ingestion for at least 4 hours before the analysis; minimal intake of 2 l of water the day before the analysis; total absence of physical activity at least 8 hours before the analysis; no drinking coffee or alcoholic beverages during at least 12 hours previous to the test; the use of diuretics was forbidden for at least 24 hours before the analysis.

The statistical analysis was carried out using the software package Statistical Package for Social Sciences ver. 19.0 (Chicago, IL, USA) considering only significance levels lower or equal to 5% (*P* ≤ 0.05). The comparison of means was carried out using the *t* test of Student for all pairwisecomparisons.

## 3. Results

The weight of the patients before the surgical procedure varied from 102.0 to 214.0 kg with an average of 139.48 ± 25.53 kg. After the procedure, the lowest weight observed among the patients was 73.0 kg while the highest was 148.0 kg. The average weight was 99.48 ± 18.27 kg. The average weight loss observed in this study was 40.00 ± 12.19 kg, representing a statistically significant (*P* < 0.001) reduction.

**Figure 1:**
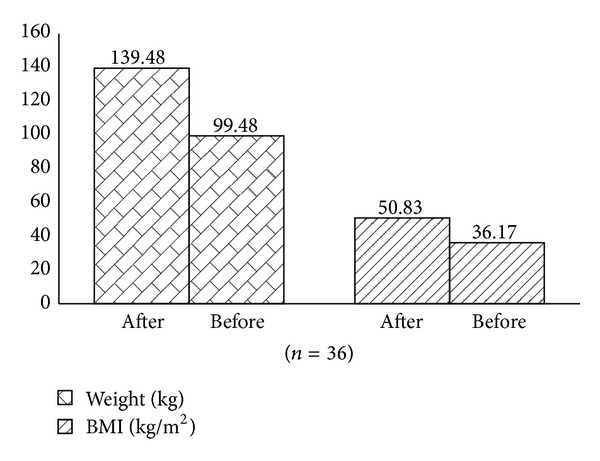
Weight and BMI before and six months after the bariatric surgery.

The BMI values before the surgery varied from 70.0 to 37.0 kg/m^2^ with an average of 50.83 ± 7.72 kg/m^2^, while after the surgery it varied from 47.0 to 27.0 kg/m^2^ with an average of 36.17 ± 5.33 kg/m^2^(Figure 1). As described for weight, the BMI also exhibited a statistical significant reduction (*P* < 0.001).

The values of total body water varied from 38.0 to 83.0 kg with an average of 50.06 ± 11.07 before the surgical procedure, while those after the surgery varied from 32.0 to 69.0 kg with an average of 44.36 ± 9.16 kg (*P* < 0.001).

In contrast, it was observed for the total body water that the percentage of body water exhibited a significant increase after the procedure varying from 29 to 48% (36.06 ± 4.79%) before the surgery and from 36 to 58% (45.00 ± 5.84%) after the surgery.

The amount of fat mass varied from 47.1 to 118.3 kg (71.00 ± 17.34 kg) before the surgery and from 20.9 to 65.0 kg (38.82 ± 12.10 kg) after the surgery. The percentage of fat mass exhibited a similar trend varying from 34.0 to 60.0% (50.67 ± 6.52%) before the surgery and from 21.0 to 49.0% (38.67 ± 7.86%) after the surgery. The reduction observed in the amount and percentage of fat mass were statistically significant (*P* < 0.001) (Table 1).

The values of fat-free mass obtained before the surgery varied from 51.70 to 112.9 kg (68.49 ± 15.03 kg) while the percentage of fat-free mass varied from 40.0 to 66.0% (49.33 ± 6.52%). Six months after the surgery, the values of fat-free mass varied from 44.2 to 94.0 kg (60.65 ± 12.49 kg) while the percentage of fat-free mass varied from 49.0 to 79.0 % (61.33 ± 7.86%).

The comparison of the average values of fat-free mass and percentage of fat-free mass before and after the surgery showed a statistically significant reduction (7.84 ± 4.38 kg) in fat-free mass and a gain (12 ± 3.91 kg) in the percentage of fat-free mass 6 months after the bariatric surgery.

From the 40.00 ± 12.19 kg lost 6 months after the surgery, 19.6% (7.84 ± 4.38 kg) corresponded to loss of fat-free mass (Table 1 and Figure 1).

The cellular mass values varied from 20.4 to 49.2 kg (29.72 ± 9.55 kg) before the surgery and from 17.3 to 43.3 kg (24.49 ± 6.86 kg) after the surgical procedure was performed.

## 4. Discussion

With the purpose of better understanding the weight variation among patients subjected to open gastric bypass, the main weight components (fat mass, fat-free mass, and body water) were assessed in 36 morbidly obese patients before and six months after the surgical procedure. The sample population involved in this study exhibited a gender distribution of 80.6% females and 19.4% males. Despite the fact that epidemiological data suggests a distribution of two obese females for each obese male, it is known that obesity level III is more prevalent among females than among males.

Regarding weight loss, it is important to emphasize that bariatric surgery resulted in an average percentage of weight loss of 28.6% (40 kg) as determined six months after the procedure was performed. These results confirm the effectiveness of open gastric bypass in the treatment of morbid obesity and support the conclusions of Buchwald et al. [1] and Sorensen [2] who suggested that the risk of surgery was lower than the risk of remaining obese and that clinical treatments exhibited very low success rates for the treatment of morbid obesity, respectively.

An important point about bariatric surgery that should be emphasized is the relationship between weight loss and the variation of the body weight components. A most desirable effect of the surgery would be the exclusive loss of fat since obesity is also defined as the presence of more than 33% of body fat with respect to total weight by SEEDO [14]. However, despite the significant loss of total weight observed in this study after the procedure, an undesirable loss of fat-free mass (about 20%) was observed six months after the surgery which may reflect a problem of malnutrition, represented by serum albumin levels lower than 3.5. This loss of fat-free mass was the same observed on another study realized to analyzing difference between an exercise and a nonexercise group by the sixth month following bariatric surgery [15].

The fat-free mass is composed of about 50% of muscle and 50% of soft tissue, bones, and collagen [16]. In this study, we excluded patients who had any complications due to the fact that electrolyte disturbance can occur which may induce errors in bioelectrical impedance analysis. The bioimpedance measuring fat mass and fat-free mass and water features relatively increase every time there is a relative increase in fat-free mass (water-rich tissue) and decrease relative fat (tissue poor in water).

The BIA is considered a highly safe method since no accidents have been reported so far even though thousands of analyses have been performed. The test is not recommended to patients carrying pacemakers since no data exists on the possible effects [12].

The BIA has several advantages over other methods since it is a noninvasive method, there is a low cost associated with it, it is easily performed, it is highly reproducible, and it is adaptable to medical routine [17]. Other studies have shown a standard error of about 9% in the determination of fat-free mass when compared to magnetic resonance [18]. When the reproducibility of the BIA was evaluated, a standard error of only 2% was detected when the same operator performed the analysis twice under the same conditions [17].

Das et al. [19] compared BIA to other commonly used tests and concluded that BIA was very good to estimate the average variation of fat-free and fat mass for populations but exhibited limitations for the analysis of individual cases. They also concluded that BIA was more reliable than BMI for the assessment of obesity regardless of the equation used [19, 20]. Therefore, it can be concluded that BIA is an appropriate test to measure obesity and mainly the body weight components [21, 22] and represents an excellent tool for monitoring patients subjected to bariatric surgery to perform nutritional evaluations [23], even when the weight loss is not complete [24].

This study reflects the loss of the first 6 months after bariatric surgery, and we intend to evaluate this group after 2 years after surgery, a period when we expect that a gain of muscle and fat-free mass would occur.

## 5. Conclusions

Open gastric bypass induced a significant loss of total weight in patients six months after the surgery. However, a significant loss of fat-free mass was also observed six months after the surgery reflecting problems of malnutrition. The use of BIA resulted in an appropriate estimation of the total weight components in individuals subjected to bariatric surgery allowing a more real analysis of the variation of weight after the surgery.

## Figures and Tables

**Table 1:** Fat mass and fat-free mass before and after bariatric surgery.

Before surgery		After surgery (6 months)	*P* value
Fat mass (kg)	71.00 ± 17.34	38.82 ± 12.10	*P* < 0.001
Fat mass (%)	50.67 ± 6.52	38.67 ± 7.86	*P* < 0.001
Fat-free mass (Kg)	68.49 ± 15.03	60.65 ± 12.49	*P* < 0.001
Fat-free mass (%)	49.33 ± 6.52	61.33 ± 7.86	*P* < 0.001
